# Convergent Regulation of Neuronal Differentiation and Erk and Akt Kinases in Human Neural Progenitor Cells by Lysophosphatidic Acid, Sphingosine 1-Phosphate, and LIF: Specific Roles for the LPA1 Receptor

**DOI:** 10.1177/1759091414558416

**Published:** 2014-11-13

**Authors:** Phillip Callihan, Mourad W. Ali, Hector Salazar, Nhat Quach, Xian Wu, Steven L. Stice, Shelley B. Hooks

**Affiliations:** 1 Department of Pharmaceutical and Biomedical Sciences, University of Georgia, Athens, GA, USA; 2 Department of Animal and Dairy Science, Regenerative Bioscience Center, University of Georgia, Athens, GA, USA

**Keywords:** lysophosphatidic acid, sphingosine 1-phosphate, neural progenitor, Akt, Erk, Ki16425, LPA1, neuronal differentiation, LIF, bFGF

## Abstract

The bioactive lysophospholipids lysophosphatidic acid (LPA) and sphingosine 1-phosphate (S1P) have diverse effects on the developing nervous system and neural progenitors, but the molecular basis for their pleiotropic effects is poorly understood. We previously defined LPA and S1P signaling in proliferating human neural progenitor (hNP) cells, and the current study investigates their role in neuronal differentiation of these cells. Differentiation in the presence of LPA or S1P significantly enhanced cell survival and decreased expression of neuronal markers. Further, the LPA receptor antagonist Ki16425 fully blocked the effects of LPA, and differentiation in the presence of Ki16425 dramatically enhanced neurite length. LPA and S1P robustly activated Erk, but surprisingly both strongly suppressed Akt activation. Ki16425 and pertussis toxin blocked LPA activation of Erk but not LPA inhibition of Akt, suggesting distinct receptor and G-protein subtypes mediate these effects. Finally, we explored cross talk between lysophospholipid signaling and the cytokine leukemia inhibitory factor (LIF). LPA/S1P effects on neuronal differentiation were amplified in the presence of LIF. Similarly, the ability of LPA/S1P to regulate Erk and Akt was impacted by the presence of LIF; LIF enhanced the inhibitory effect of LPA/S1P on Akt phosphorylation, while LIF blunted the activation of Erk by LPA/S1P. Taken together, our results suggest that LPA and S1P enhance survival and inhibit neuronal differentiation of hNP cells, and LPA1 is critical for the effect of LPA. The pleiotropic effects of LPA may reflect differences in receptor subtype expression or cross talk with LIF receptor signaling.

## Introduction

Lysophosphatidic acid (LPA) and sphingosine 1-phosphate (S1P) are lysophospholipid ligands for G-protein coupled receptors (GPCRs) that regulate cell growth, survival, morphology, and gene expression in cancer and development. Lysophospholipids and their receptors have critical and diverse effects on stem cell and neural progenitor cell populations, and their effects in these systems have been well reviewed (Pebay et al., 2007; Pitson and Pebay, 2009). LPA and S1P are endogenously generated in the developing brain by the enzymes autotaxin and sphingosine kinases, respectively, and multiple LPA and S1P receptors are expressed in neural progenitors and neurons (Fukushima et al., 2000; Birgbauer and Chun, 2006; Kimura et al., 2007). Endogenous LPA and S1P are critical in early neural tube development and neural progenitor survival and proliferation. Genetic deletion of sphingosine kinases or autotaxin results in neural tube defects with high levels of apoptotic cells in the neural progenitor cell layer (Mizugishi et al., 2005; van Meeteren et al., 2006). In more recent complimentary studies, mice lacking lipid phosphatase enzymes that degrade endogenous LPA and S1P show loss of neuronal differentiation and neurite outgrowth (Sanchez-Sanchez et al., 2012). These results demonstrate critical roles for endogenous LPA and S1P in promoting progenitor cell survival and suppressing neuronal differentiation *in vivo*.

Multiple studies have attempted to delineate the mechanisms underlying the effects of LPA, and to a lesser extent S1P, on neural progenitor biology *in vitro*. However, a clear role for LPA has not emerged due to pleiotropic effects, variable receptor expression profiles, and species differences in LPA effects. LPA has broad effects on proliferation, survival, morphology, and differentiation in multiple neural stem/progenitor models, with distinct effects on neural stem/progenitor cells (NS/PCs) derived from different sources (Daub et al., 1997; Fukushima et al., 2000; Gschwind et al., 2002; Kue et al., 2002; Harada et al., 2004; Pebay et al., 2005; Cui and Qiao, 2006; Dottori et al., 2008). In multiple rodent-derived models, LPA promotes neuronal differentiation. For example, in rat embryonic cortical neural stem cell cultures, LPA promotes proliferation and the formation of Map2 and choline acetyltransferase (ChAT)-positive neurons and enhances neurite extension (Cui and Qiao, 2006). In mouse neural progenitors, LPA blocks neurosphere growth but promotes neuronal differentiation through LPA-Gi-mediated pathways (Fukushima et al., 2007). In contrast to its effect on rodent neural progenitors, in human embryonic stem cell (hESC)-derived NS/PCs grown as neurospheres, LPA inhibits neuronal differentiation, blocking neurosphere formation and expression of neuronal markers (Dottori et al., 2008; Frisca et al., 2013). The molecular basis for the opposing effects of LPA in these different systems is unknown but may reflect distinct expression profiles of LPA receptors or other signaling pathway regulators that intersect LPA receptor signaling. Less is known about the effects of S1P on neural progenitor biology, but S1P did not affect neuronal differentiation in human NS/PC cells (Pitson and Pebay, 2009).

LPA and S1P mediate their effects by binding and activating multiple GPCRs. S1P activates five related members of the endothelial differentiation gene (Edg) family of receptors (S1P1-5), while LPA activates three receptors from the Edg gene family (LPA1-3), and several additional LPA receptors related to purinergic receptors (LPA4-6; Tabata et al., 2007; Murakami et al., 2008; Pasternack et al., 2008). LPA receptors each couple to multiple G-protein pathways; LPA1, 2, and 5 can couple to Gi, Gq, and G12, while LPA3 couples to Gi and Gq, but not G12, and LPA4 and LPA6 couple to all major G-protein families including Gs (Im et al., 2000). Similarly, S1P1 receptor subtype couples only to Gi, while S1P2 and S1P3 couple to Gi, Gq, and G12 type G-proteins, and S1P4 and S1P5 couple to Gi and G12 (Maceyka et al., 2009). Thus, the specific complement of receptor subtypes expressed in a given cell type will dramatically alter the global effect of LPA or S1P on cell signaling cascades. Activation of Gs stimulates cAMP synthesis, while Gi activation is associated with inhibition of cAMP and activation of the Ras/Erk Map kinase and phosphoinositide-3 kinase (PI3K)/Akt kinase pathways. Signaling through Gq activates primarily Phospholipase C pathways, while signaling through G12 primarily activates Rho and its target kinase ROCK (Hurst et al., 2008a). There is significant cross talk between these pathways (Katz et al., 1992; Maier et al., 1999), and the presence of additional receptors that impact these pathways may dictate the effect of LPA and S1P in a given system. For example, the cytokine leukemia inhibitory factor (LIF) and the growth factor basic fibroblast growth factor (bFGF) regulate Akt and Erk signaling cascades, allowing potential cross talk with LPA and S1P signaling (see Discussion section).

Our laboratory has characterized LPA and S1P signaling pathways in hESC-derived neural progenitors (hNPs; termed hES-NEP cells in a previous publication (Hurst et al., 2008b)) and defined their effects on hNP cell proliferation and morphology (Hurst et al., 2008b). hNP cells were derived from ES cell aggregates in the presence of bFGF and express high levels of neural progenitor markers Sox2 and Nestin and low Oct-4 expression (Shin et al., 2006). hNP cells maintain this phenotype over multiple passages when cultured in the presence of bFGF. hNP cells express multiple LPA and S1P receptors and LPA and S1P trigger multiple signaling cascades in these cells (Callihan et al., 2010). Briefly, LPA and S1P inhibit cAMP accumulation via Gi and activate Gq-like activation of PLC activity in hNP cells (Hurst et al., 2008b).We have also shown that LPA and S1P stimulate Gi- and Erk-dependent hNP cell proliferation and stimulate Rho kinase-dependent, Gi-independent neurite retraction in hNP cells. Thus, multiple signaling pathways are activated in response to LPA and S1P in proliferating hNP cells cultured in the presence of bFGF (Hurst et al., 2008a). hNP cells undergo spontaneous differentiation to a predominantly neuronal cell type on withdrawal of bFGF; however, the role of LPA and S1P in regulating neuronal differentiation of these cells has not been described.

Neural progenitor cells are critical in the development of the central nervous system and in maintaining tissue homeostasis in the adult brain (Vaccarino et al., 2001; Gotz and Huttner, 2005). Neurogenesis is regulated *in vivo* by endogenous biochemical cues, including LPA, S1P, and multiple kinase coupled receptor ligands, which together dictate whether neural progenitors continue to proliferate and maintain the stem cell population, or differentiate into neurons or glial cells (Harada et al., 2004; Pebay et al., 2005; Cui and Qiao, 2006; Dottori et al., 2008). Manipulating neural progenitors to stimulate neurogenesis *in vivo* or *in vitro* holds significant therapeutic potential in reversing the loss of neurons through either neurodegenerative disease or injury. However, to harness this therapeutic potential, it is critical to define the molecular mechanisms by which endogenous biochemical cues regulate receptor signaling pathways to instruct neural stem cells to differentiate, especially in the context of complex mixtures of growth factors as they exist *in vivo*. The lysophospholipids LPA and S1P are endogenously generated ligands for GPCRs with important roles in regulating neural stem cell growth, differentiation, and migration toward sites of injury. However, they activate multiple receptors with overlapping, sometimes opposing functions, and LPA has been reported to have distinct effects in different neural stem cell models. This study demonstrates that lysophospholipids enhance survival and inhibit neuronal differentiation of hNP cells, with unexpected mechanisms and synergy with the cytokine LIF.

## Materials and Methods

### hNP Cell Culture

Commercially available stocks of hNP cells were obtained from Aruna Biomedical (STEMEZ™ hNP1™, Aruna Biomedical). Tissue culture plates were coated with matrigel (BD Biosciences) diluted 1:200 in NEUROBASAL™ medium (GIBCO) for 1.5 hr at room temperature and washed with HyClone® DPBS/MODIFIED (1×) with calcium and magnesium (PBS^++^; Thermo Scientific) prior to application of media containing cells. Cells were grown in hNP proliferation media, AB2™ media with ANS™ (Aruna Biomedical) supplemented with 2 mM l-glutamine (Sigma) and 20 ng/mL b-FGF (R&D Systems), in the absence of LIF. Cells were passaged approximately every 48 hr and split 1:2 following manual dissociation using a 25-cm cell scraper (Sarstedt).

### hNP Cell Differentiation

hNP cells were plated in a T-75 flask (50% confluent) coated with matrigel and were incubated for 24 hr at 37℃. hNP proliferation media were aspirated and replaced with 10 mL of differentiation media lacking bFGF. In indicated differentiation experiments, media were supplemented with 10 ng/mL LIF. hNP cells were incubated for 72 hr at 37℃. All media were aspirated from each T-75 flask and were replaced with fresh media. Cells were removed from plate using a soft edge cell scraper and plated in 96-well matrigel-coated plates at 30,000 cells/well or six-well matrigel-coated plates at 500,000 cells/well. Cells were incubated for 24 hr at 37℃, at which point the indicated drug was added to each well. All media and drug were aspirated every 72 hr and replaced with fresh media/drug. Cells were grown in hNP differentiation media for a total of 14 days prior to assay.

### Cellular Viability Assays

Approximately 30,000 hNP cells per well were differentiated in matrigel-coated 96-well plates and treated with the indicated concentrations of LPA, S1P, or other drugs. A cell viability assay was conducted by adding 7 µL of CellTiter-Blue® reagent (Promega Corporation), incubated at 37℃ for 4 hr, and measured using SpectraMax M2 model microplate reader (Molecular Devices).

### Cell Fixing, Staining, and Imaging

One hundred microliters of warm fixation solution containing 8% paraformaldehyde/8% sucrose in PBS^++^ were added to each well of a 96-well plate. Plates were incubated for 20 min at 37℃. All media and fixation solution were aspirated, and cells were washed once with 200 µL warm PBS^++^. Cells were then washed three times with 100 µL of Block/Permeabilization buffer containing 1% BSA/0.1% saponin in PBS^++^. Fifty microliters of primary antibody solution containing primary antibody, 1% BSA, and 0.02% Na Azide in PBS^++^ were added to each well. The following primary antibody dilutions were used: 1:50 Rabbit anti-Ki67 (Abcam); 1:200 Rabbit anti-Map2 (Neuromics); 1:200 Mouse anti-Tubulin (βIII-tubulin, Tuj; Neuromics). Plates were incubated at room temperature for 1 hr. All primary antibody solution was aspirated from each well, and cells were washed twice with 100 µL PBS^++^. Then, 50 µL of secondary antibody solution containing secondary antibody, 3 µg/mL DAPI, and 1% BSA in PBS^++^ were added to each well. The following secondary antibody dilutions were used: 1:1,000 Donkey anti-Rabbit Alexa Fluor® 488 conjugated (Invitrogen); 1:1,000 Goat anti-Mouse Alexa Fluor® 488 conjugated (Invitrogen). Cells were incubated in secondary antibody solution for 1 hr at room temperature. All secondary antibody solution was aspirated from each well, and cells were washed twice with 100 µL PBS^++^. Two hundred microliters of PBS^++^ were added to each well, and plates were sealed with optical plate covers.

Cells were imaged using the CellomicsThermo Scientific ArrayScan VTI HCS reader. The Cellomics platform was used to obtain 20 images per well of cells plated in 96-well plates. Image analysis was performed using the Cell Health Profiling BioApplication version 4 to assess total live cell count and using the Neuronal Profiling BioApplication version 4 algorithms to assess Map2-positive cells, tubulin-positive cells, and neurite extension. Nuclear size and border shape exclusion parameters were used to exclude condensed, apoptotic nuclei according to the manufacturer’s optimization protocols; these algorithms have been widely used and validated (Marteyn et al., 2011; Robinette et al., 2011). Ki67 expression was assessed using a modified version of the standard profiling algorithm. Briefly, Cellomics HCS software algorithms were optimized and used to identify DAPI-stained nuclei in Channel 1. Channel 2 staining associated with each nucleus was quantified, and all cells displaying Channel 2 intensity over a set threshold were considered positive for Ki-67. Cells were stained with DAPI and Ki67 primary antibody with secondary fluorescent antibody (emission: 519). Cells were imaged using a Cellomics imaging system algorithm which simultaneously images DAPI and Ki67 (Figure 3(a)). DAPI staining was used by the algorithm to identify and count valid nuclei. Ki67 staining intensity was quantified, and cells above a set threshold (which is optimized using the control reference wells) scored as positive for Ki-67.

### Western Blot Analysis

Cells were plated at 80,000 cells/well in 24-well plates coated with matrigel and were incubated for 24 hr at 37℃. hNP cell medium was aspirated and replaced with 0.5 mL of media lacking bFGF, and cells were incubated for 18 hr at 37℃. Then, 50 µL of 10× drug were added to each well, and the cells were incubated for 10 or 30 min at 37℃. The reaction was terminated by aspirating the media and adding 100 µL SDS-PAGE sample buffer. Cells lysates were boiled for 5 min in protein sample buffer, separated by SDS-PAGE, transferred to nitrocellulose membranes, and immunoblotted using primary antibodies targeted against phosphoSer473 Akt, or phosphop42/44 Erk1/2 MapKinase(Cell Signaling Technologies) and peroxidase-conjugated secondary antibody (Bethyl Laboratories). Bands were visualized using SuperSignal Chemiluminescent substrate (Pierce). Densitometry analysis was performed using Alpha InnotechFluorchem® HD2 software. Densitometry results were normalized to GAPDH to control for loading.

### Quantitative Real-Time Polymerase Chain Reaction

After differentiating and dosing hNP cells in six-well plates, Trizol reagent (Invitrogen) was added. RNA isolation was performed according to the manufacturer’s protocol. DNA was synthesized from 2 µg of total RNA using the High Capacity Reverse Transcriptase cDNA kit (Applied Biosystems) to amplify the mRNA. Following cDNA synthesis, quantitative real-time polymerase chain reaction was performed using Superscript III kit for real-time polymerase chain reaction (RT-PCR; Invitrogen) and Master Mix containing Power SYBR Green reagent (Applied Biosystems). Transcript expression was assessed using a 7900HT RT-PCR System from Applied Biosystems (Life Technologies). Reactions were normalized using the housekeeping gene β2 microglobulin, and calculations were performed according to the 2^−ΔΔCT^ method.

### Statistical Analysis

Data were analyzed for variance using ANOVA, and differences were determined using an unpaired, two-tailed *t* test. Details on the statistical methods used for each experiment are included in the figure legends. *p* values less than .05 were considered significant. * indicates *p* < .05, ** indicates *p* < .01, and *** indicates *p* < .001.

## Results

### LIF, LPA, and S1P Promote Cell Survival During *In Vitro* Neuronal Differentiation

Withdrawal of bFGF inhibits proliferation and induces cell death in the majority of hNP cells; however, a fraction of cells survive and those that do undergo terminal differentiation toward a predominantly (∼95%) neuronal cell fate (Shin et al., 2006; Dhara and Stice, 2008). To determine the effects of LPA and S1P on cell survival during neuronal differentiation, hNP cells were differentiated via bFGF withdrawal for 14 days. LPA (1 µM) or S1P (0.1 µM) was added to differentiating cells for the final 10 days of differentiation. These optimal LPA and S1P concentrations were determined based on previous dose-response curves in hNP cells (Hurst et al., 2008b). Prior to differentiation, hNP cells were maintained in the absence of LIF, and the effect of LIF on survival during differentiation was determined by adding 10 ng/mL LIF to cells at the time of bFGF withdrawal. Cell survival was measured at the end of the 14-day differentiation by the complimentary approaches of automated counting of DAPI-stained cells and by assessing cell viability using the mitochondrial metabolism indicator CellTiter Blue.

**Figure 1.**
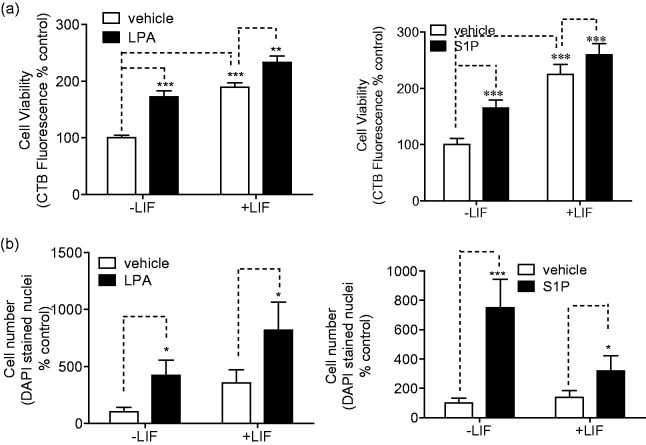
LPA and S1P promote hNP cell survival during differentiation. Cells were differentiated by 14-day bFGF withdrawal as described in Materials and Methods section in the presence or absence of 1 µM LPA, 0.1 µM S1P, or 10 ng/mL LIF. Cell viability and counting assays were carried out on day 14. Data are reported as percentage of vehicle-treated, LIF control wells. (a) Cell viability was assessed using CellTiter-Blue® mitochondrial metabolism reagent as described. Results are representative of three independent experiments. (b) Cell number was determined by counting DAPI-stained nuclei using Cellomics automated image analysis, as described.

LPA and S1P significantly increased cell survival as measured by cell number or cell viability, and their effects are approximately additive with LIF effects (Figure 1). Notably, LIF treatment alone increased cell viability as measured by metabolism indicators, but did not increase the number of surviving cells, suggesting a possible increase in cell metabolic activity. To determine whether the observed increases in cell viability and cell number were due to increases in the number of proliferating cells, we measured the expression of the proliferation marker Ki-67 after differentiation. Ki-67 is expressed in the nucleus of proliferating cells during all stages of the cell cycle but not in quiescent cells (Scholzen and Gerdes, 2000). Characteristic punctuate nuclear Ki67 staining was observed in the hNP cells grown in the presence of bFGF, and Ki67 expression was significantly decreased in cells differentiated by 2 weeks of bFGF withdrawal. However, the presence of LIF, S1P, or LPA during differentiation did not affect Ki-67 expression compared with control-differentiated cells (data not shown).These results are consistent with our recent report that LIF blocks caspase activation and apoptosis in hNP cells following bFGF withdrawal (Majumder et al., 2012) and suggest that the LPA- and S1P-induced increases in cell metabolism and cell number during differentiation are due to increased cell survival, not increased cell proliferation.

### LPA and S1P Effects on Neuronal Differentiation: Expression of Neuronal Proteins

**Figure 2.**
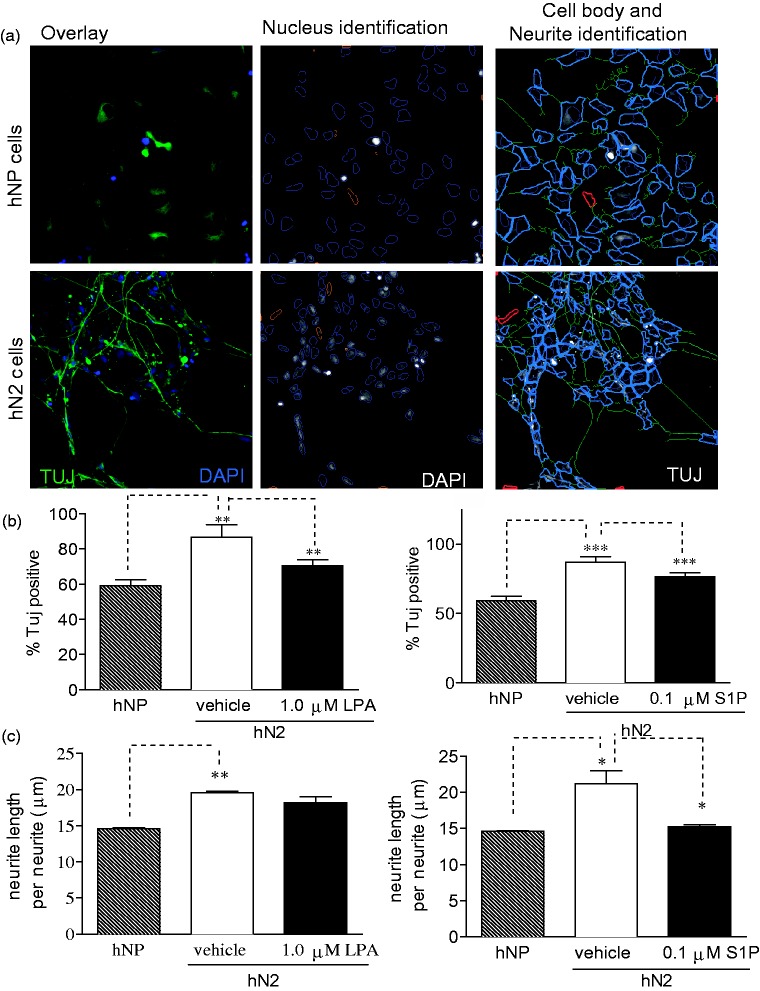
LPA and S1P suppress expression of the neuronal marker βIII-tubulin during *in vitro* differentiation. Cells were differentiated for 14 days in the absence of bFGF and treated with LPA or S1P as indicated. Cells were fixed, stained, imaged, and analyzed as described in Materials and Methods section. (a) Neuronal profiling algorithm for quantification of βIII-tubulin (Tuj) staining. Upper panels: Undifferentiated hNP cells grown in the presence of bFGF. Lower panels: hN2 cells differentiated by withdrawal of bFGF for 14 days. Left panels: Overlay of DAPI-stained nuclei (blue) and tubulin staining in cell bodies and neurites (green). Middle panels: Nucleus identification algorithm image analysis. Objects outlined in blue were identified as valid nuclei and used for further analysis, while objects outlined in orange were rejected based on algorithm criteria for size, shape, intensity, and image border-intersecting criteria. Right panels: Cell body identification and quantification and neurite identification and measurement algorithm image analysis. Cell bodies were identified based on tubulin staining intensity (shown in grayscale); objects outlined in light blue represent a cell body associated with an identified nucleus. Cells with tubulin staining intensity above a set threshold were scored as positive for tubulin expression. Excluded cell bodies are shown in red. Neurites are identified in green tracing and measured by Cellomics neuronal profiling algorithm. (b) Tubulin expression levels are reported as a percentage of cells expressing tubulin above a set threshold. NP: hNP cells grown in the presence of bFGF. N2: hN2 cells differentiated via bFGF for 2 weeks. (c) Average neurite length of cells treated as indicated.

**Figure 3.**
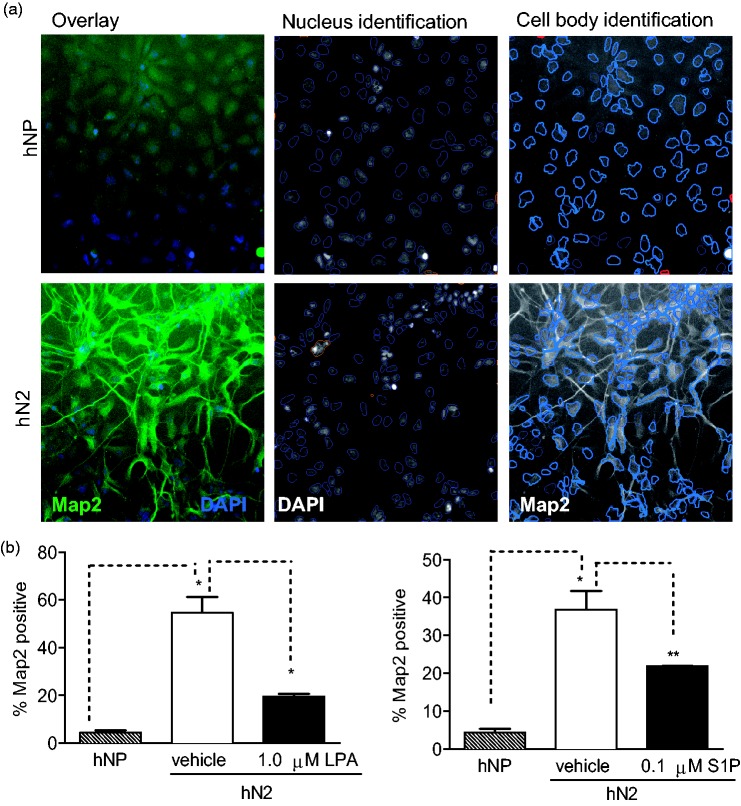
LPA and S1P suppress expression of the neuronal marker Map2 during *in vitro* differentiation. Cells were differentiated and analyzed as described in Materials and Methods section and Figure 2. (a) Neuronal profiling algorithm for quantification of Map2 staining. Upper panels: Undifferentiated hNP cells grown in the presence of bFGF. Lower panels: hN2 cells differentiated by withdrawal of bFGF for 14 days. Left panels: Overlay of DAPI-stained nuclei (blue) and Map2 staining in cell bodies and neurites (green). Middle panels: Nucleus identification algorithm image analysis. Objects outlined in blue were identified as nuclei and used for further analysis, while objects outlined in orange were rejected based on size, shape, intensity, and image border-intersecting criteria. Right panels: Cell body identification and quantification and neurite identification and measurement algorithm image analysis. Cell bodies were identified based on Map2 staining intensity (shown in grayscale); objects outlined in light blue represent a cell body associated with an identified nucleus. Cells with Map2 staining intensity above a set threshold were scored as positive for Map2 expression. Excluded cell bodies are shown in red. Neurites are identified in green tracing and measured by Cellomics neuronal profiling algorithm. (b) Map2 expression levels are reported as a percentage of cells expressing Map2 above a set threshold. NP: hNP cells grown in the presence of bFGF. N2: hN2 cells differentiated via bFGF for 2 weeks.

Following 2 weeks of bFGF withdrawal, the majority of hNP cells lose expression of Nestin and Sox-2, induce expression neuronal markers βIII-tubulin and Map2, and form extensive neurite outgrowth (Dhara and Stice, 2008). To determine the effect of LPA and S1P on *in vitro* neuronal differentiation of hNP cells, we measured the levels of βIII-tubulin in cells differentiated in the presence or absence of LPA or S1P using Cellomics High Content Screening. Expression of βIII-tubulin (Tuj) was detected at moderate levels in the undifferentiated hNP cells, but expression was significantly higher in control cells differentiated by bFGF withdrawal for 2 weeks (hN2; Figure 2). Differentiation in the presence of 1 µM LPA or 0.1 µM S1P significantly inhibited expression of βIII-tubulin compared with control-differentiated hN2 cells. We performed the same analysis for expression of microtubule-associated protein 2 (Map2), a marker for later neurons, following 2-week bFGF withdrawal (Figure 3). The expression of Map2 was very low in undifferentiated hNP cells, and expression was markedly higher in control-differentiated hN2 cells. Consistent with tubulin-stained cells, expression of Map2 was dramatically inhibited by the presence of LPA or S1P during differentiation via bFGF withdrawal. Data are shown as a percent of total surviving cells; LPA and S1P also decreased the total number of Tuj- or MAP2-postive cells (data not shown).

### LPA and S1P Effects on Neuronal Differentiation: Neurite Extension

As hNP cells differentiate, they undergo distinct morphological changes that include the formation and elongation of neurites (Sanes et al., 2006). Previous reports indicate that LPA and S1P regulate neuronal morphology and neurite biology (Jalink et al., 1993; Sato et al., 1997; Fukushima et al., 2002), and our laboratory has shown that these lysophospholipids induce reversible cell rounding of hNP cells (Hurst et al., 2008a). Cellomics Neural Profiling algorithm was used to count neurites and measure neurite length in hNP cells differentiated in the presence of LPA or S1P and labeled with βIII-tubulin antibodies. Undifferentiated hNP cells display many small extensions, while control-differentiated hN2 cells show fewer but significantly longer neurites. Differentiation in the presence of S1P significantly reduced neurite length (Figure 2(c)). We consistently observed a modest decrease in neurite length in cells differentiated in the presence of LPA, but the effects were not statistically significant.

### Effect of LPA1 Receptor Selective Antagonist on Neuronal Differentiation

hNP cells express multiple LPA receptor isoforms; specifically, quantitative RT-PCR (qRT-PCR) data show expression of LPA1>LPA2≫LPA4>LPA5 with undetectable levels of LPA3 (Hurst et al., 2008a). Given that LPA1 is expressed at highest levels, we predicted that this receptor is critical in mediating the observed effects of LPA on the differentiation of hNP cells. To test this prediction, we differentiated cells in the presence of the receptor antagonists Ki16425. Ki16425 is a dual LPA1/3 antagonist, but as hNP cells do not express LPA3, Ki16425 functions as an LPA1 receptor specific antagonist in these cells (Ohta et al., 2003). Cell survival, neuronal marker expression, and neurite extension were assessed after the 14-day differentiation in the presence of 10 µM Ki16425 alone or in the presence of 1 µM LPA.

**Figure 4.**
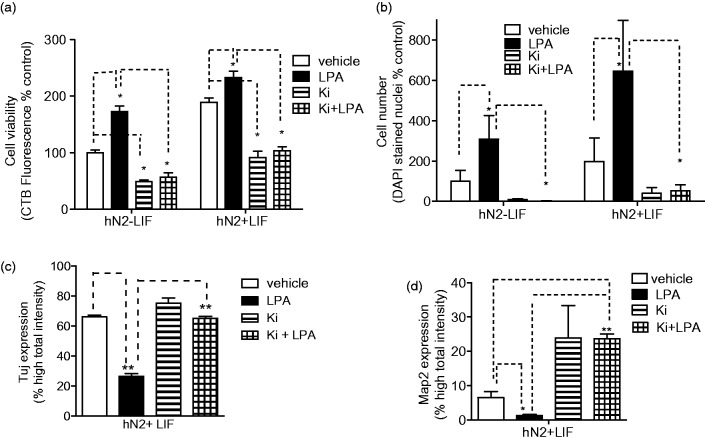
LPA1 receptor selective antagonist Ki16425 inhibits LPA effects on differentiation of hNP cells. hNP cells were differentiated for 14 days, in the presence of 1 µM LPA or 10 µM Ki16425 for the final 10 days. Cell survival and protein expression were assessed as described. Cell viability (a) and DAPI-stained nucleus count (b) are reported as % of vehicle-treated, LIF control wells. βIII-tubulin (Tuj) expression (c) and Map2 expression (d) are reported as percent of differentiated cells expressing each marker above a set threshold.

**Figure 5.**
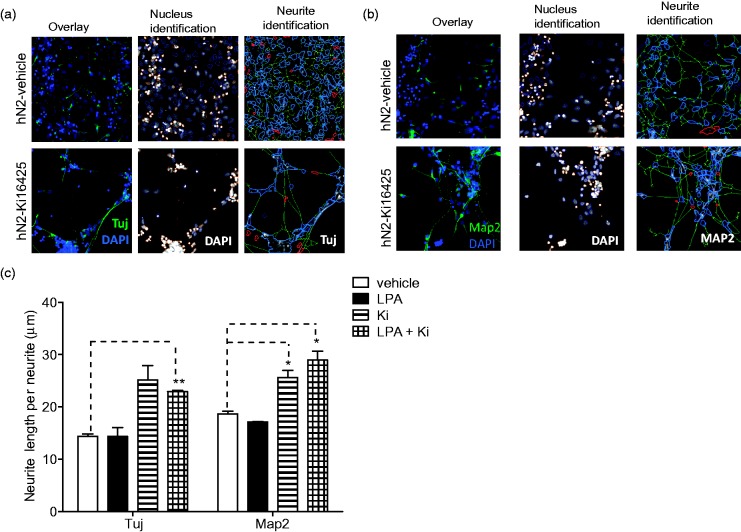
Ki16425 increases length of neurites in differentiated neurons. hNP cells were differentiated for 14 days, in the presence of 1 µM LPA or 10 µM Ki16425 for the final 10 days. Cells were fixed and stained with βIII-tubulin (Tuj) or Map2 as described, and neurite length was quantified using Cellomics neuronal profiling algorithm. (a) Left panels: Overlay of DAPI-stained nuclei (blue) and tubulin staining (green). Middle panels: Nucleus identification algorithm image analysis. Objects outlined in blue were identified as nuclei and used for further analysis, while objects outlined in orange were rejected based on size, shape, intensity, and image border-intersecting criteria. Right panels: Neurite identification algorithm image analysis. Neurites are identified in green tracing and measured by Cellomics neuronal profiling algorithm. (b) Map2 immunofluorescence staining. (c) The average length of neurites in nhN2 cells differentiated under the indicated conditions as shown.

Differentiation in the presence of Ki16425 dramatically affected cell survival and neuronal differentiation. The presence of LPA during differentiation increased cell survival as before, and this survival effect was completely inhibited by 10 µM Ki16425, suggesting a requirement for LPA1 in mediating this effect. Further, Ki16425 treatment alone decreased hNP cell survival below vehicle-treated levels in the presence and absence of LPA (Figure 4), suggesting that autocrine or constitutive signaling through LPA1 may promote hNP cell survival during differentiation. We next determined the effect of Ki16425 on LPA suppression of the neuronal markers βIII-tubulin and Map2. As seen previously, the presence of LPA during differentiation inhibited βIII-tubulin expression, but cotreatment with the LPA1 blocker Ki16425 completely blocked the inhibitory effect of exogenous LPA on neuronal differentiation. Similarly, Ki16425 completely blocked the ability of LPA to inhibit Map2 expression and also enhanced neuronal Map2 expression above levels seen in control-differentiated cells (Figure 4). Finally, striking differences in cell morphology were observed in cells differentiated in the presence of Ki16425. Neurite length was quantified in cells differentiated in the presence of LPA or Ki16425 using the Cellomics neuronal profiling algorithm as described. Neurite length was markedly higher in cells differentiated in the presence of Ki16425 in cells stained with either βIII-tubulin or Map2 (Figure 5). Notably, Ki16425 effects on neurite length occurred in the presence or absence of exogenous LPA. These results suggest that LPA stimulates LPA1-mediated pathways that oppose neuronal differentiation and selective inhibition of LPA1 receptors may significantly enhance *in vitro* neuronal differentiation of hNPs.

We similarly explored the roles of S1P1 and S1P3 receptors in mediating the effects of S1P on neuronal differentiation by differentiating hNP cells in the presence of the receptor antagonist VPC23019, which inhibits the activity of both S1P1 and S1P3 receptors (Davis et al., 2005). VPC23019 did not have a significant effect on S1P regulation of hNP cell survival or neuronal differentiation and was not assessed further (data not shown, see Discussion section).

### LPA and S1P Regulation of Akt and Erk Phosphorylation

**Figure 6.**
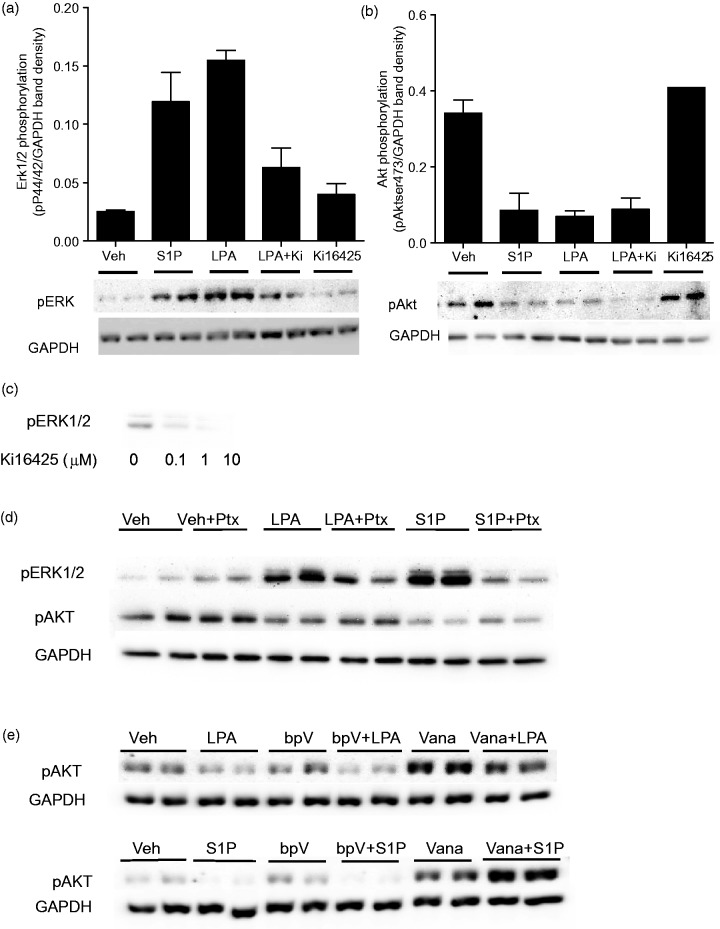
LPA and S1P decrease Akt phosphorylation and increase Erk phosphorylation in hNP cells. hNP cells were grown in the absence of bFGF for 24 hr and treated with the indicated compounds in fresh media. (a) Cells were treated with 0.1 µM S1P, 1 µM LPA, or 10 µM Ki16425 for 10 min and then harvested for Western blotting analysis with anti-phospho p42/44 Erk Map kinase antibodies. Expression of GAPDH protein was determined as a housekeeping standard. Band intensities were quantified and Erk phosphorylation levels were normalized to GAPDH levels. (b) Cells were treated with 0.1 µM S1P, 1 µM LPA, or 10 µM Ki16425 for 30 minutes, and then harvested for Western blotting analysis with anti-phospho serine 473 Akt kinase antibodies. Expression of GAPDH protein was determined as a housekeeping standard. Band intensities were quantified and Erk phosphorylation levels were normalized to GAPDH levels. (c) hNP cells were grown in the absence of bFGF for 24 hr and treated with the indicated concentrations of Ki16425 in the absence of exogenous LPA for 10 minutes. Cells were harvested and analyzed by Western blotting analysis with anti-phospho p42/44 Erk Map kinase antibodies. (d) hNP cells were treated in duplicate with 1 µM LPA or 0.1 µM S1P with or without pretreatment with 100 ng/mL pertussis toxin (Ptx) for 12 hr. ERK1/2 phosphorylation was assessed after 10-min LPA/S1P treatment, and Akt phosphorylation was assessed after 30-min LPA/S1P treatment. (e) hNP cells were treated in duplicate with 1 µM LPA or 0.1 µM S1P with or without 30-min pretreatment with 2.5 µM bpV(OHpic) or 100 µM sodium vanadate. ERK1/2 phosphorylation was assessed after 10-min LPA/S1P treatment, and Akt phosphorylation was assessed after 30-min LPA/S1P treatment.

Akt and Map kinase signaling pathways are established mediators of self-renewal and differentiation pathways (Watanabe et al., 2006; Li et al., 2007), and both LPA and S1P activate these kinase pathways in multiple systems (Cook et al., 1997; Baudhuin et al., 2002; Ye et al., 2002; Yu et al., 2004). Our laboratory has previously shown that LPA and S1P induce Erk Map kinase phosphorylation in proliferating hNP cells grown in the presence of bFGF (Hurst et al., 2008b). To determine the effects of LPA and S1P on Akt and Erk phosphorylation under differentiation conditions, hNP cells were bFGF-starved for 24 hr and then treated with LPA or S1P for 10 min to assess Erk activation and for 30 min to assess Akt activation. Cell media were replaced at the time of LPA/S1P addition, to remove any endogenously generated mediators. As expected, LPA and S1P stimulated Erk1/2 phosphorylation. Surprisingly, LPA and S1P both inhibited phosphorylation at Thr 308 and Ser 473 activation sites on Akt (shown for Ser473, Figure 6(a) and (b)). These results demonstrate that LPA and S1P have opposing effects on Erk and Akt signaling in hNP cells and strongly alter the balance of Akt and Erk signaling.

Given Ki16425 effects on neuronal differentiation, we next determined the ability of this receptor antagonist to block the distinct effects of LPA on Erk and Akt activation. Ki16425 fully blocked LPA-stimulated Erk phosphorylation, indicating that this is an LPA1-mediated effect. Strikingly, Ki16425 had no effect on LPA-stimulated decreases in Akt phosphorylation in hNP cells, suggesting that this effect is mediated by a distinct receptor subtype. Further, as we observed effects of Ki16425 on neuronal differentiation in the absence of exogenous LPA, we predicted that Ki16425 may inhibit LPA1 constitutive activity or activation by endogenous LPA. To test this possibility, we determined the effect of Ki16425 on Erk Map kinase activity in cells maintained in conditioned media with no added LPA. Under these conditions, basal levels of phosphorylated Map kinase are clearly detectable. Treatment of hNP cells with Ki16425 for 10 min showed dose-dependent inhibition of basal Erk phosphorylation in the absence of exogenous LPA, consistent with the presence of autocrine activation LPA1 activity in hNP cells (Figure 6(c)).

We next sought to determine the relative role of Gi-coupled LPA and S1P receptors in the opposing effects on Erk and Akt pathways. We observed that pretreatment of hNP cells with the Gi-selective inhibitor pertussis toxin (Ptx) abolished the activation of Erk1/2 phosphorylation by LPA/S1P but had no effect on LPA-/S1P-stimulated suppression of Akt phosphorylation (Figure 6(d)). Akt phosphorylation is dynamically regulated by the opposing actions of kinases including PI3 kinase (PI3K) and phosphatases. Further, PI3K activation is dynamically regulated by multiple activators and the inhibitory enzyme PTEN. While LPA and S1P typically activate Akt phosphorylation via a PI3K-dependent activity, we predicted that in hNP cells, LPA and S1P may inhibit Akt phosphorylation by activating either PTEN or general phosphatase activity. To test these possibilities, we determined the ability of LPA and S1P to suppress Akt phosphorylation in the presence of the PTEN inhibitor bpv(OHpic) and the general phosphatase inhibitor sodium vanadate. Inhibition of PTEN did not block the ability of LPA or S1P to inhibit Akt phosphorylation. However, vanadate treatment blunted the ability of LPA to suppress Akt phosphorylation and completely blocked the ability of S1P to suppress Akt phosphorylation. In fact, in the presence of vanadate, S1P enhanced Akt phosphorylation (Figure 6(e)). Thus, S1P-mediated inhibition of Akt phosphorylation requires phosphatase activity, which masks the canonical activation of Akt phosphorylation by S1P.

### bFGF and LIF Impacts on LPA and S1P Regulation of Akt and Erk Phosphorylation

**Figure 7.**
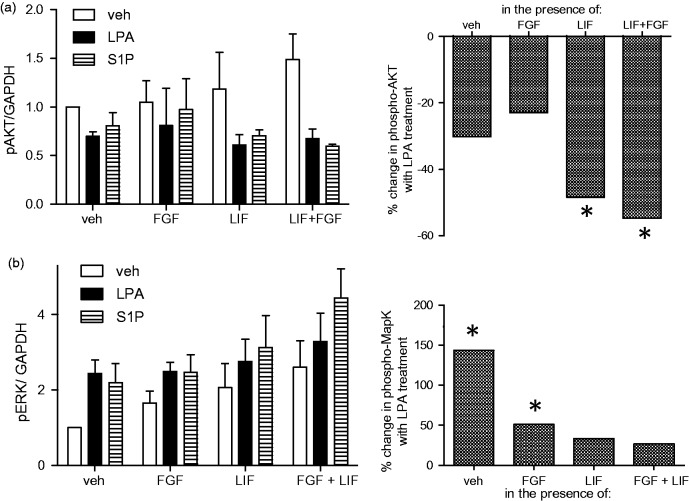
Costimulation with LIF and bFGF alter the effect of LPA and S1P on Erk and Akt signaling pathways. hNP cells were grown in the absence of bFGF for 24 hr and were then stimulated with 20 ng/mL bFGF, 10 ng/mL LIF, 1 µM LPA, or 0.1 µM S1P as indicated. (a) To assess Erk Map kinase activation, cells were treated for 10 min and then harvested for Western blotting analysis with anti-phospho p42/44 Erk Map kinase antibodies. Expression of GAPDH protein was determined as a housekeeping standard. Band intensities were quantified and Erk phosphorylation levels were normalized to GAPDH levels. (b) To assess Akt activation, cells were treated for 30 min and then harvested for Western blotting analysis with anti-phospho serine 473 Akt kinase antibodies. Expression of GAPDH protein was determined as a housekeeping standard. Band intensities were quantified and Erk phosphorylation levels were normalized to GAPDH levels. Right panels: The ability of LPA to regulate Erk and Akt activation levels was compared when added to cells alone or in the presence of bFGF, LIF, or LIF and bFGF. Under each condition, the percent change in phosphorylation in LPA-treated cells versus vehicle controls was determined. *p < .05.

LPA effects on neuronal differentiation are strikingly different in neural progenitors derived from human and rodent sources, and similarly, the cytokine LIF and the growth factor bFGF are known to have different effects in human and rodent stem cells (see Discussion section). Given that bFGF and LIF signaling pathways intersect LPA signaling pathways via regulation of Erk and Akt, we predicted that convergent regulation of these pathways may contribute to the pleiotropic effects of LPA. Thus, we directly tested the ability of LIF and bFGF to influence the effect of LPA and S1P on Erk and Akt activity. Each of the compounds was added individually and in combination to hNP cells grown for 24 hr in the absence of all factors. The activation of Erk and Akt was measured following 10 or 30 min treatment, respectively. When administered individually, LPA and S1P inhibited Akt phosphorylation and enhanced Erk phosphorylation, while LIF and bFGF treatment increased both Akt and Erk activation (Figure 7). Thus, LPA and S1P mimic the effects of LIF and bFGF on Erk activation and oppose the effects of LIF and bFGF on Akt activation. When combined, LIF and bFGF enhanced the apparent impact of LPA or S1P on Akt by elevating activation in the absence of LPA/S1P, while the ability of LIF/bFGF to saturate Erk Map kinase activation masked the apparent impact of LPA and S1P on Erk Map kinase. Therefore, coactivation of LIF receptors with LPA/S1P receptors may significantly impact their apparent effect on neural progenitor cell signaling.

### LIF Impacts on LPA and S1P Regulation of Neuronal Marker Expression

**Figure 8.**
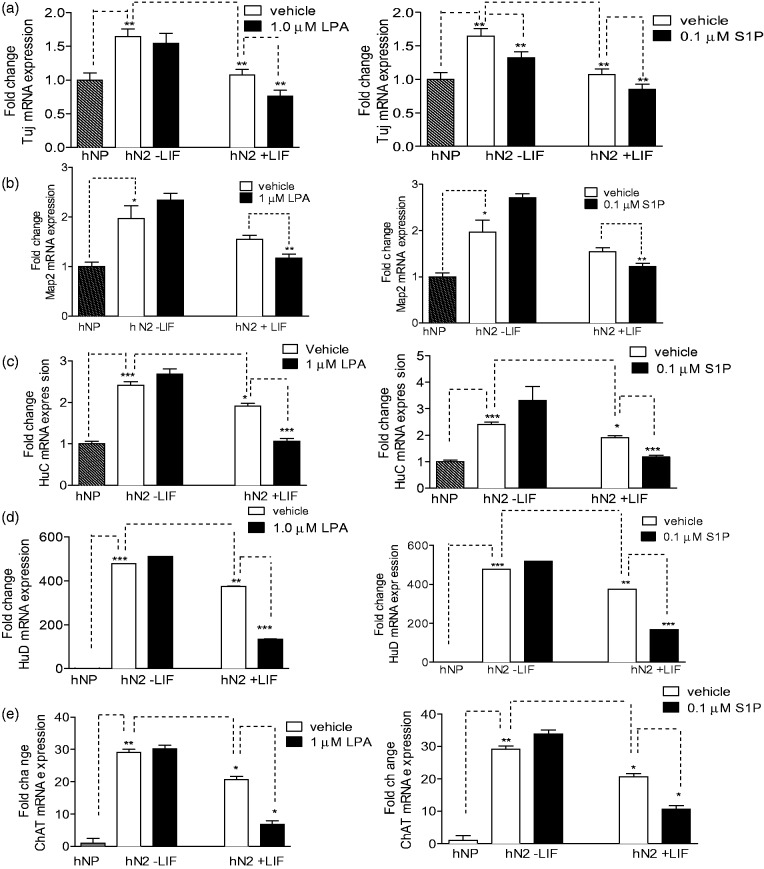
LPA and S1P inhibit mRNA expression of neuronal markers. hNP cells were differentiated by bFGF withdrawal for 2 weeks treated with LPA, S1P, or LIF, and qPCR analysis was conducted as described in Materials and Methods section. Data are reported as fold change in expression compared with hNP cells grown in bFGF containing media (NP). (a) βIII-tubulin mRNA expression. (b) Map2 mRNA expression. (c) HuC mRNA expression. (d) HuD mRNA expression. (e) ChAT mRNA expression. *p < .05. **p < .01. ***p < .001.

Next we assessed the impact of LIF signaling on the ability of LPA and S1P to inhibit neuronal differentiation of hNP cells. We performed qRT-PCR to measure mRNA transcripts of neuronal markers in hNP cells differentiated by bFGF withdrawal in the presence or absence of LPA, S1P, and LIF. Consistent with protein expression results, βIII-tubulin and Map2 mRNA levels were higher in differentiated neurons than in neural progenitors, but lower in cells differentiated in the presence of LPA or S1P as compared with control-differentiated cells. However, suppression of Map2 transcript by LPA or S1P was only observed in the presence of LIF (Figure 8). To determine if this effect was unique to Map2, we measured expression additional neuronal markers in hNP cells differentiated under these conditions: HuC and HuD and neuron-specific members of the ELAV family of RNA-binding proteins (Perrone-Bizzozero and Bolognani, 2002), and ChAT is a marker of cholinergic neurons (Cui and Qiao, 2006). Differentiation via bFGF withdrawal dramatically upregulated HuC, HuD and ChAT as expected. Similar to the Map2 mRNA expression pattern, differentiation in the presence of LPA or S1P significantly inhibited expression of HuC, HuD, and ChAT only in the presence of LIF (Figure 8). Thus, the ability of LPA to inhibit expression of certain neuronal markers may require or be synergistic with LIF receptor activation. These data suggest that differential activity of LIF pathways may in part account for the different effects of LPA on expression of neuronal markers such as ChAT in different neural progenitor systems (Cui and Qiao, 2006).

## Discussion

The goal of the current study was to define the ability of LPA and S1P to regulate neuronal differentiation of hNP cells on bFGF withdrawal and to define cross talk between bFGF and LIF signaling pathways. We report that in this system, both LPA and S1P enhance hNP survival during *in vitro* differentiation and inhibit expression of neuronal markers. We also identified specific roles of the LPA1 receptor subtype, and cross talk between LPA, S1P, LIF, and bFGF pathways in the regulation of Erk and Akt kinase activation. These studies demonstrate that LPA and S1P inhibit neuronal differentiation of hNPs and suggest that the pleiotropic effects of LPA on progenitor cells from different sources may reflect differences in LPA1 receptor expression or interactions with cytokine pathways.

A surprising result in this study is that LPA and S1P have opposing effects on Akt and Erk signaling pathways. Strikingly, we demonstrate that LPA and S1P *inhibit* Akt activation in these cells, while activating Erk phosphorylation. While LPA and S1P have been reported to activate Akt in numerous systems including many neural cell types (Ye et al., 2002; Kim et al., 2008), Hla and colleagues have described opposing effects of S1P on Akt signaling in Human umbilical vein endothelial cells (HUVECs). Specifically, they report S1P activation of Akt phosphorylation via S1P1, while S1P2 opposed Akt activation via a PTEN-dependent mechanism (Sanchez et al., 2005, 2007). Thus, we predicted that S1P inhibition of Akt signaling in hNP cells would be similarly PTEN dependent. However, we observed reversal of the S1P effect on Akt signaling following inhibition of phosphatases enzymes, but not following inhibition of PTEN. Further work is needed to define the mechanism and receptor selectivity of LPA and S1P regulation of Akt signaling in hNP cells. Regardless, our results suggest that the activation of LPA and S1P strongly alters the balance of Akt and Erk signaling by inhibiting Akt and promoting Erk. This could tip the balance in favor of Erk pathways known to regulate cell survival and growth, while also blocking Akt signaling pathways that regulate differentiation.

Our data suggest that the LPA receptor LPA1 is critical in mediating LPA effects on neural progenitor survival and neuronal marker expression. We also observed that Ki16425 inhibited hNP survival and promoted neurite extension in the absence of exogenous LPA, suggesting a possible endogenous tone of LPA1 receptor activity via autocrine LPA production or LPA1 constitutive activity. The ability of acute Ki16425 treatment to suppress basal Erk Map kinase phosphorylation supports this possibility. The inhibitor used is a potent and selective inhibitor of LPA1 receptors; however, the potential for off-target effects of pharmacological inhibitors remains. Unfortunately, attempts to confirm these data with LPA1 receptor knockdown were unsuccessful due to low transient transfection rates, and the growth inhibitory effects of loss of LPA1 prevented stable line selection. Thus, additional studies are needed to fully define the role of the LPA1 receptor subtype in the observed effects of LPA. Further, we show that the LPA1 receptor is critical for LPA-mediated increases in Erk activation but is not required for suppression of Akt activity. Similarly, Ptx blocks LPA- and S1P-mediated increases in Erk phosphorylation but does not affect the inhibition of Akt. These results suggest that the relative activation of strongly Gi-coupled receptors such as LPA1 versus Gi-independent receptor pathways may dictate the ultimate effect of LPA and S1P on neural progenitors, and the effects on Erk and Akt signaling pathways will vary significantly in cells with distinct receptor expression profiles.

Our results in hNP cells are generally consistent with previous reports that LPA inhibits neuronal differentiation in human neural stem/progenitor cells (hNS/PCs) cultured in neurospheres (Dottori et al., 2008; Frisca et al., 2013), but key differences were also observed. First, we observed similar effects for both LPA and S1P in hNP cells, while S1P did not have an effect on hNS/PCs (Pitson and Pebay, 2009). hNS/PCs were reported to lack S1P2 expression while hNP cells express S1P2, which may account for the ability of S1P to regulate hNP cells. Indeed, the effects of S1P were not consistently blocked using the S1P1/S1P3 antagonist VPC23019 (data not shown). Future studies using S1P2 selective ligands may address the specific function of this receptor subtype in hNP cells. Second, Frisca et al. (2013) report that 10 µM LPA inhibited proliferation and stimulated apoptosis in hNS/PC cells, seemingly in contradiction to the LPA-stimulated increases in cell growth reported herein. Importantly, our results were observed with 1 µM LPA; we also observed increased hNP cell death with LPA concentrations above 10 µM (data not shown). Similar biphasic effects on cell growth have been widely reported for LPA (Robinson-White et al., 2003; Gustin et al., 2008). A final distinction between LPA effects in hNP cells and hNS/PCs is the involvement of the LPA1 receptor subtype. The ability of the LPA1/3 selective antagonist Ki16425 to completely block both the survival effects of LPA and the inhibition of neuronal marker expression by LPA suggest a predominant role for this receptor subtype in mediating the effects of LPA in differentiating hNP cells. In contrast, inhibition of proliferation in NS/PCs mediated by 10 µM LPA was independent of the LPA1 receptor subtype and required Rho/ROCK (Frisca et al., 2013). It is possible that a higher potency, LPA1-mediated survival effect is masked by a lower potency Rho-mediated, LPA1-independent proapoptotic effect at higher concentrations of LPA in hNS/PC cells. Indeed, the relative expression of LPA1 is higher in hNP cells than in NS/PCs. Further, 18:1 LPA (the species used in these studies) has a higher affinity for LPA1 receptors than LPA2-5 (Choi et al., 2010). Thus, LPA1 receptor signaling likely predominates in hNP cells. Future studies are needed to define the role of Rho/ROCK pathways, which are critical in mediating neuronal differentiation in NS/PCs, in the effects of LPA, and the LPA1 antagonist in hNP cells (Frisca et al., 2013).

Our results contrast reports that LPA promotes neuronal differentiation in rodent models. Notably, rat neural progenitor/stem cells express high levels of LPA1 and LPA3, with only weak expression of LPA2 (Cui and Qiao, 2007), while hNPs express high levels of LPA1 and LPA2, with undetectable levels of LPA3 (Hurst et al., 2008b). The G-protein coupling of LPA1, 2, and 3 is distinct. LPA1 preferentially couples to Gi (An et al., 1998), while LPA2 has stronger coupling to Gq and G12 G-proteins (Bandoh et al., 1999). LPA3 preferentially couples to Gi and Gq, and not G12 (Im et al., 2000). Thus, distinct receptor expression levels in rat and hNPs may contribute to the observed differences. Further, the hNP cell system used here is highly purified and homogeneous, while rodent cultures are a heterogeneous culture of multiple cell types.

Finally, another possible explanation for the pleiotropic effects of LPA on neural progenitor/stem cells from different systems is that LPA signaling overlaps significantly with signaling mediated by bFGF and LIF growth factors, which also have variable effects in different systems. The predominant target of FGF receptor activation is Erk Map kinase (Dvorak et al., 2005; Cai et al., 2010), but bFGF also triggers activation of PI3K/Akt signaling, which contributes to bFGF-mediated pluripotency (Dvorak et al., 2005; Ding et al., 2010). LIF is a member of the IL-6 family of cytokines and regulates survival and differentiation of multiple stem cell populations. The canonical activity of LIF is mediated through activation of a heteromeric receptor composed of gp130 and the LIF receptor (LIFR; Ernst and Jenkins, 2004) to activate Janus kinase (Jak) to phosphorylate its major target, signal transducer and activator of transcription (Stat; Matsuda et al., 1999; Niwa et al., 2009). LIF also activates the PI3K-Akt pathway (Niwa et al., 2009) and Erk Map kinase cascades (Ying et al., 2008). LIF and bFGF have distinct effects on mouse and human embryonic stem cells (ESCs). LIF is a key pluripotency factor in maintaining mouse ESCs via activation of PI3K (Smith et al., 1988; Williams et al., 1988), but LIF is not sufficient to maintain pluripotency in human ESCs (Ginis et al., 2004; Wei et al., 2005).

bFGF and LIF also have complex roles in regulating neural progenitor cells. FGFs play important roles in the survival and expansion of neural progenitors (Wilson et al., 2000), and the ERK Map kinase pathway is specifically implicated in the maintenance of adult neural progenitor proliferation (Ma et al., 2009). LIF has contradictory effects on NS/PC differentiation: directly promoting astrocyte differentiation while reducing neuronal differentiation (Bonni et al., 1997), promoting neural stem cell maintenance (Hatta et al., 2002), or enhancing neuronal differentiation (Richards et al., 1996). We have recently reported that LIF enhances neuronal survival of differentiating hNP cells (Majumder et al., 2012). A critical difference in the cell culture conditions in that study was the maintenance of hNP cells in the presence of LIF prior to differentiation; in the current study, hNP cells were maintained in the absence of LIF until initiation of differentiation. We have shown that culturing hNP cells in the presence of LIF upregulates expression of the LIF coreceptor gp130 (Majumder et al., 2012), suggesting that chronic exposure to LIF may alter LIF signaling. Additional studies are needed to further define the effect of LIF on hNP neuronal differentiation and survival.

Our results demonstrate that lysophospholipid signaling converges with LIF- and bFGF-mediated signaling to coregulate Erk and Akt kinases. These results may in part explain conflicting reports of the role of LPA signaling in different neural progenitor populations. In particular, LPA has been reported to inhibit neuronal differentiation of hNPs through activation of PI3K and Rho (Dottori et al., 2008), which is very different from the inhibition of Akt observed here. It is possible that the interaction of multiple inputs converging on kinases cascades such as Akt and Erk contributes to these discrepancies, and specifically that the ability of LPA (and S1P) to significantly inhibit Akt phosphorylation requires high basal Akt activation, such as in the presence of LIF or FGF, and may require specific signaling cascades that couple G-proteins to phosphatase regulation.

The ability of LPA and S1P to regulate neural progenitor growth and differentiation has significant implications in tissue regeneration and recovery following injury. Both lysophospholipids are released from activated platelets during injury, and LPA and S1P production rises dramatically in the brain following injury or ischemia (Tigyi et al., 1995; Savaskan et al., 2007; Kimura et al., 2008). Blocking endogenous LPA with neutralizing antibodies in animal models of spinal cord injury significantly enhances neuronal survival and recovery (Goldshmit et al., 2012), and the S1P2 receptor antagonist enhances the migration of transplanted neural progenitors to site of spinal cord injury (Kimura et al., 2008). These findings suggest that blocking receptor activation by endogenous LPA and S1P may be critical to the successful mobilization of either endogenous or exogenous neural progenitors for therapeutic recovery from neural injury or neurodegenerative disease. Our results further suggest that effective therapeutic manipulation of these receptors will require defining the roles of specific lysophospholipid receptor subtypes, which may have opposing functions, and defining the impact of additional receptor pathways that may amplify or blunt the functions of LPA and S1P.

## Summary

This study explores the molecular mechanisms by which bioactive lipids regulate neuronal differentiation of human neural progenitors. These lipids inhibit neuronal differentiation, unexpectedly inhibit Akt signaling, and exhibit synergistic interactions with cytokine signaling pathways.
